# Stability, reliability, and validity of the THINC‐it screening tool for cognitive impairment in depression: A psychometric exploration in healthy volunteers

**DOI:** 10.1002/mpr.1736

**Published:** 2018-08-07

**Authors:** John E. Harrison, Harry Barry, Bernhard T. Baune, Michael W. Best, Christopher R. Bowie, Danielle S. Cha, Larry Culpepper, Philippe Fossati, Tracy L. Greer, Catherine Harmer, Esther Klag, Raymond W. Lam, Yena Lee, Rodrigo B. Mansur, Hans‐Ulrich Wittchen, Roger S. McIntyre

**Affiliations:** ^1^ Alzheimer Center VU Medical Center Amsterdam The Netherlands; ^2^ Department of Psychological Medicine, Institute of Psychiatry, Psychology and Neuroscience King's College London London UK; ^3^ Kilmington Common Metis Cognition Ltd Wiltshire UK; ^4^ Stoneylane Clinic Drogheda Co Louth Ireland; ^5^ Discipline of Psychiatry University of Adelaide Adelaide Australia; ^6^ Department of Psychology Queen's University Kingston ON Canada; ^7^ Centre for Addiction and Mental Health Toronto ON Canada; ^8^ Mood Disorders Psychopharmacology Unit University Health Network Toronto ON Canada; ^9^ Department of Family Medicine Boston University Boston Massachusetts USA; ^10^ Sorbonne Universités, UPMC Univ Paris 06, Inserm, CNRS, APHP Institut du cerveau et de la moelle (ICM)‐Hôpital Pitié Salpétrière Paris France; ^11^ Centre for Depression Research and Clinical Care, Department of Psychiatry University of Texas Dallas Texas USA; ^12^ Cognitive Neuroscience, Department of Psychiatry Oxford University Oxford UK; ^13^ Clinical Psychologist, Rehabilitation Neuropsychologist, Psychotherapist London UK; ^14^ BC Leadership Chair in Depression Research Vancouver BC Canada; ^15^ Department of Psychiatry University of British Columbia Vancouver BC Canada; ^16^ Djavad Mowafaghian Centre for Brain Health Mood Disorders Centre Vancouver BC Canada; ^17^ Canadian Network for Mood and Anxiety Treatments (CANMAT) Vancouver BC Canada; ^18^ Institute of Medical Science University of Toronto Toronto ON Canada; ^19^ Institute of Clinical Psychology and Psychotherapy and Centre of Clinical Epidemiology and Longitudinal Studies, (CELOS) Technische Universität Dresden Dresden Germany; ^20^ Department of Psychiatry and Psychotherapy Ludwig‐Maximilians‐University Munich Munich Germany; ^21^ Clinical Psychology and Psychotherapy MPCB Dresden Germany; ^22^ Department of Psychiatry University of Toronto Toronto ON Canada; ^23^ Brain and Cognition Discovery Foundation (BCDF) Toronto ON Canada; ^24^ Department of Pharmacology University of Toronto Toronto ON Canada

**Keywords:** cognition, depression, memory, neuropsychological, screening

## Abstract

**Objectives:**

There is a need for a brief, reliable, valid, and sensitive assessment tool for screening cognitive deficits in patients with Major Depressive Disorders. This paper examines the psychometric characteristics of THINC‐it, a cognitive assessment tool composed of four objective measures of cognition and a self‐rated assessment, in subjects without mental disorders.

**Methods:**

*N* = 100 healthy controls with no current or past history of depression were tested on four sequential assessments to examine temporal stability, reliability, and convergent validity of the THINC‐it tests. We examined temporal reliability across 1 week and stability via three consecutive assessments. Consistency of assessment by the study rater (intrarater reliability) was calculated using the data from the second and third of these consecutive assessments.

**Results:**

Test–retest reliability correlations varied between Pearson's *r* = 0.75 and 0.8. Intrarater reliability between 0.7 and 0.93. Stability for the primary measure for each test yielded within‐subject standard deviation values between 5.9 and 11.23 for accuracy measures and 0.735 and 17.3 seconds for latency measures. Convergent validity for three tasks was in the acceptable range, but low for the Symbol Check task.

**Conclusions:**

Analysis shows high levels of reliability and stability. Levels of convergent validity were modest but acceptable in the case of all but one test.

## 1 INTRODUCTION

Recent research into the cognitive difficulties experienced by patients with Major Depressive Disorders (MDD) has revealed a reduced capacity in function equal to an effect size in the region of 0.5 across various cognitive domains when compared to the performance of typical controls, even when patients are in remission (e.g., Rock, Roiser, Riedel, & Blackwell, 2014). These cognitive difficulties have been shown to be present at the first episode of depression (Lee, Hermens, Porter, & Redoblado‐Hodge, 2012), and a significant number of patients continue to experience difficulties between depressive episodes (Conradi, Ormel, & de Jonge, 2011; Roca et al., 2015).

Clinical research has yielded various candidate measures for assessing, evaluating, and detecting cognitive difficulties in patients with MDD (see Harrison, Lam, Baune, & McIntyre, 2016). This literature has been helpful for identifying the cognitive domains in which depression associated impairment is observed, as well as the magnitude of these effects. This research has also been instrumental to inform the selection of cognitive assessments appropriate for screening purposes for cognitive difficulties in patients with MDD.

Routine care screening for cognitive deficits in patients with depression remains a rare phenomenon, unless other nonmood diagnoses are contemplated (McAllister‐Williams et al., 2017). This is especially the case in older patients, in whom dementia might be suspected. When cognitive performance is assessed, it is typically with brief, portmanteau tests such as the Mini‐Mental States Examination and the Montreal Cognitive Assessment (Folstein, Folstein, & McHugh, 1975; Nasreddine et al., 2005). Both measures have a useful role to play as brief, bedside tests of global cognitive function, but are very unlikely to detect the types, forms, and the severity of cognitive dysfunction seen in MDD. Standardized tests for measuring cognitive difficulties in patients with MDD would offer health care professionals a further option for assessment. The demands of contemporary patient care require that screening measures and tests of change in the patients' status should be tested using reliable, sensitive, and valid measures (Harrison, 2016). Other computerized cognitive measures have been employed to assess the cognitive performance of patients with MDD. For example, the Cambridge Neuropsychological Test Automated Battery system has been employed in several studies, the results of which were recently reported in a meta‐analysis (Rock et al., 2014). Additionally, both the CogState system (Harrison & Maruff, 2008) and the assessment from central nervous system vital signs (Gualtieri & Johnson, 2006) have been employed to investigate cognitive function in depression. These systems contain computerized paradigms designed to index key cognitive areas. However, a challenge to employing one of these proprietary testing platforms is the task duration. Potential clinical users informed us that a brief assessment was required. Candidate tests of, for example, executive functions, can be rather lengthy. For example, the Groton Maze Learning Test requires at least 7 minutes (https://www.cogstate.com/cognitive-tests/groton-maze-learning/). Cambridge Neuropsychological Test Automated Battery measures, such as the “One Touch Stockings of Cambridge” Test, require at least 10 minutes to administer (http://www.cambridgecognition.com/cantab/cognitive-tests/executive-function/one-touch-stockings-of-cambridge-ots/). A further issue mitigating against the use of these systems was our need to provide tests for free.

Recently, we have developed and field‐tested a novel screening tool for health care professionals called THINC‐it that assesses key domains of function known to be compromised in patients with MDD. THINC‐it is a digital, gamified cognitive assessment tool developed by the THINC Task Force (http://thinc.progress.im), composed of experts in psychology, psychiatry, primary care, psychometrics, neuroscience, and scale development. The THINC‐it tool is accessed via computers/tablets and is composed of well‐known cognitive paradigms. The selected paradigms were chosen on the basis of their prior use with patients with MDD and their brevity. A further selection principal was to employ paradigms that are acknowledged to index performance in the key cognitive areas of working memory, attention, and executive function. The “One‐Back” paradigm (Kirchner, 1958) was selected as a measure of working memory (“Symbol Check”); Choice Reaction Time (Donders, 1969) as the measure of attention (“Spotter”), and Part B of the Trail Making Test (Strauss, Sherman, & Spreen, 2006) as a measure of executive function (“Trails”). In addition to these paradigms, it was also decided to include a computerized variant (“Codebreaker”) of the Digit Symbol Substitution Test (DSST) paradigm (Lezak, 1995). Competent DSST performance is considered dependent on the functional integrity of various cognitive skills, including working memory, attention, and executive function (Harrison, Lophaven, & Olsen, 2016). In addition to these four objective measures of cognition, THINC‐it also includes the Perceived Deficits Questionnaire—Depression, 5‐item (PDQ‐5‐D) as a subjective measure of cognitive function. This measure asks the patient to rate his/her performance regarding attention/concentration, planning/organization, and retrospective and prospective memory (Lam et al., 2013; Lovera et al., 2006). Each of the THINC‐it assessments have been employed in studies involving adults with MDD and evaluate domains of cognitive function affected in MDD (McIntyre et al., 2017) Validation reports of the individual objective measures of cognition contained within THINC‐it are published elsewhere. These tests have been shown to be sensitive to cognitive deficits in MDD and are independent of cultural background (McIntyre et al., 2017).

Recently, we reported initial evidence of THINC‐it feasibility, “caseness” and the ability to differentiate MDD patients from healthy controls (McIntyre et al., 2017). In this present report, we examine

1. the temporal reliability of the THINC‐it tests across a one‐week period;
2. the within‐subject variance of THINC‐it test performance as a measure of test stability;
3. the correlation of the THINC‐it paradigms measures with analogues of the selected tasks; and
4. the intrarater reliability of the measures.

## 2 METHODS

### 2.1 Design

**Figure 1:**
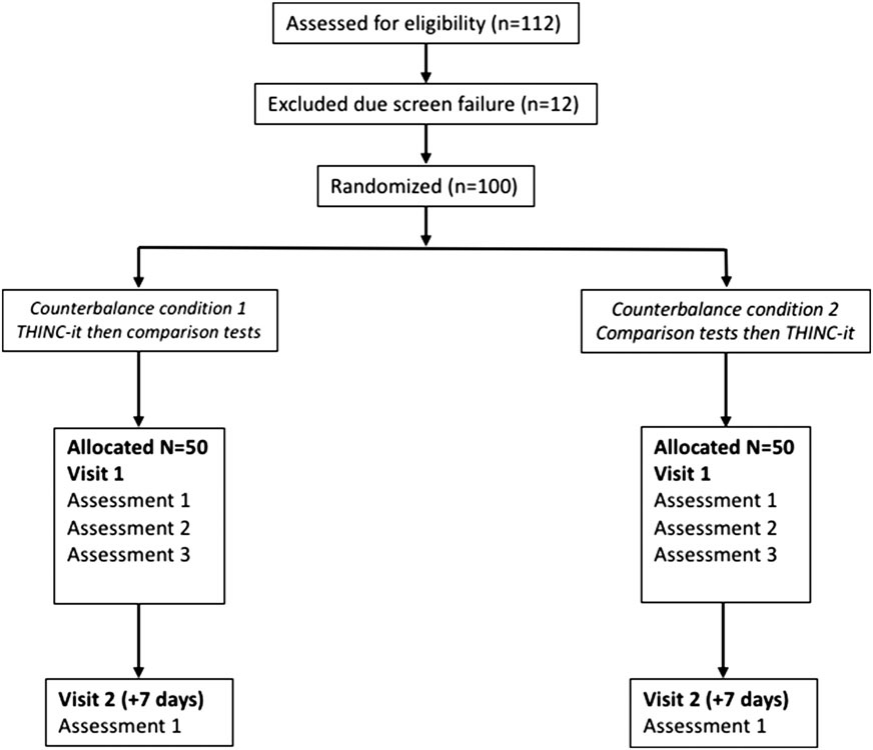
Flow chart of the study design

This analysis was part of a larger and more comprehensive project in which the cognitive performance of patients with MDD was assessed using a complex study and testing protocol (McIntyre et al., 2017). The study design and a summary of visits and assessments is shown in Figure 1. Full details of the trial are reported at https://clinicaltrials.gov/ct2/show/NCT02508493. Briefly, the first study visit was planned to allow for correlations between THINC‐it and comparison tests to determine levels of concurrent validity. Calculations of within‐subject standard deviation (WSD) across all four THINC‐it assessments at Visit 1 allowed for estimates of test stability to be made. Correlations between Visit 1 Assessments 2 and 3 were designed to determine the levels of intrarater reliability. Study Visit 2 was incorporated to evaluate estimates of temporal reliability which were determined by correlating performance.

### 2.2 Study participants

The *N* = 100 subjects were recruited via social media. They responded to media announcements seeking healthy controls who wished to participate in a study evaluating cognitive function. The media website was http://www.kijiji.com. All subjects were carefully screened and examined for current and past mental disorder by the Mini International Neuropsychiatric Interview for Diagnostic and Statistical Manual of Mental Disorders (Sheehan et al., 2006). Healthy subjects were included if they had no current nor a past mental disorder, no first‐degree relative with an established diagnosis of a lifetime mood or psychiatric disorder. Exclusion criteria were (a) unstable medical disorder(s) (b) any medication that, in the opinion of the investigator, may have affected cognitive function (e.g., corticosteroids and beta‐blockers); and (c) consumption of alcohol within 8 hr prior to the THINC‐it tool administration. Participants received financial compensation for their participation of 50.00 CAD per visit.

### 2.3 Assessments

The assessment instruments featured the iPad version of the THINC‐it tool (i.e., Spotter, Symbol Check, Codebreaker, Trails, and PDQ‐5‐D); the Identification Task and One‐Back Memory (OBK) task from the CogState battery, as well as the pencil‐and‐paper versions of the DSST, Trail Making Test Part B (TMT‐B) and PDQ‐5‐D. The National Adult Reading Test—Revised was also included as an estimate premorbid IQ (Nelson, 1982). For each THINC‐it test, a primary measure was selected for analysis. We selected the measures analogous to those most typically chosen for the CogState and paper‐and‐pencil measures. These measures are reported in Table 1. THINC‐it takes approximately 15 min to administer with instructions commensurate with minimal education for administration (i.e., Grade 6).

**Table 1:** THINC‐it tasks and details of primary metrics for each test

THINC‐it test	Comparison test	Measure
Spotter	Identification	Mean latency for correct responses as expressed in msec.^a^
Symbol Check	One‐Back Test	Number of correct responses
Codebreaker	DSST^b^	Number of correct responses
Trails	Trail Making Test Part B	Time taken for completion

a Responses quicker than 100 ms were treated as anticipations.

b Digit Symbol Substitution Test.

### 2.4 Procedures

The order of administration between the THINC‐it tool and the comparison tests (the two CogState tasks and pencil‐and‐paper test versions) was alternated between study participants to account for potential order effects. The sequence of the THINC‐it tool component scales remained identical for all study participants and was administered in the following order: Spotter, Symbol Check, Codebreaker, Trails, and PDQ‐5‐D. The comparison tests were administered in the following order: Identification Task, OBK, DSST, TMT‐B, and PDQ‐5‐D. All participants completed the full set of cognitive assessments (i.e., THINC‐it tool, CogState, and pencil‐and‐paper tests) on four occasions: three times during the first visit and once during the second visit (see Figure 1 for details of study flow).

## 3 RESULTS

The demographics and clinical characteristics of our study participants are presented in Table 2. The mean age of the 58 females and 42 male subjects was 40 (*SD* = 14.4). The group was relatively well educated and exhibited a National Adult Reading Test—Revised IQ estimate equal to a full‐scale IQ of 111.87 (*SD* = 6.63). Data for *n* = 8 healthy controls were excluded for those that did not complete the THINC‐it assessment in its entirety. The primary reasons were the subject's inability or unwillingness to complete the tasks.

**Table 2:** Demographic details of study cohort

Characteristic	Results
Age, years (mean [SD])	39.98 (14.38)
Gender, *n* (%)	
Female	58 (58.00)
Male	42 (42.00)
Ethnicity, *n* (%)	
Caucasian	56 (56.00)
Black	9 (9.00)
Hawaiian	1 (1.00)
Asian	31 (31.00)
Native American	0 (0.00)
South Asian	3 (3.00)
Education, years (from Grade 1; mean [SD])	16.26 (2.73)
MADRS^a^ score (mean [SD])	0.77 (1.42)
NART‐R^b^ full‐scale IQ	111.87 (6.63)

a Montgomery*‐*Åsberg Depression Rating Scale.

b National Adult Reading Test (revised).

Median and mean values for all THINC‐it and comparison tasks were found to be similar and so we report only means and confidence intervals (CI) based on standard errors of the mean. As the data met the requirements for parametric analysis, we calculated Pearson's “*r*” correlations in all cases. These values are reported for each THINC‐it test measure and as a composite of all four tests, by Visit and Assessment, in Table 3, together with the same statistics for all four paper‐and‐pencil versions of the selected paradigms, as well as for a composite score of all four THINC‐it tasks.

**Table 3:** Mean and 95% CI (based on standard error of the mean) for objective THINC‐it cognitive measures and their near equivalents by Visit (V) and Assessment (A)

Test	Visit1 (V1) Assessment 1 (A1)	V1_A2	V1_A3	V2	IRR^a^ (95% CI)
Spotter (mean latency for correct responses)	655 (616–693)	595 (562–629)	569 (539–599)	577 (545–609)	0.93 [0.90, 0.95]
Symbol Check (number correct)	21 (19–23)	27 (24–29)	28 (26–31)	29 (27–31)	0.91 [0.87, 0.94]
Codebreaker (number of correct responses)	55 (51–58)	65 (62–68)	69 (66–73)	69 (65–73)	0.79 [0.7, 0.85]
Trails (time taken for completion)	42 (35–48)	27 (24–31)	27 (21–32)	27 (23–32)	0.70 [0.58, .79]
Mean of all THINC‐it tasks	0.46 (0.45–0.47)	0.49 (0.48–0.50)	0.5 (.49–0.51)	0.5 (0.49–0.51)	0.94 [0.91, 0.96]
Identification (mean latency in ms log10)	2.77 (2.75–2.8)	2.8 (2.75–2.85)	2.77 (2.75–2.79)	2.76 (2.74–2.78)	N/A
One‐Back Task (number correct)	28 (26.2–28.8)	29 (27.6–29.7)	29 (27.9–30.1)	29 (27.6–29.8)	N/A
DSST (number of correct responses)	62 (59–66)	67 (64–70)	70 (67–74)	72 (68–75)	N/A
TMT‐B^b^ (time taken for completion)	78 (68–89)	64 (58–71)	61 (54–68)	56 (51–61)	N/A

a Intrarater reliability (IRR), calculated as the *r*‐value correlation between V1_A2 and V1_A3.

b Trail Making Test Part B.

*Temporal*, or “test–retest,” *reliability* was calculated by correlating Visit 1 Assessment 1 scores with Visit 2 scores, which were separated for 1 week. This analysis yielded Pearson's “*r*” correlations for the four objective measures varying between 0.74 and 0.81 (all significant at <0.001) and a value of 0.72 for the PDQ‐D‐5 (see Table 4). These correlations were higher when comparisons were made between Visit 1 Assessment 3 and Visit 2. This is likely due to greater performance stability, suggesting some repeated exposure effects. We report also the temporal reliability correlation between Visit 1 Assessments 2 and 3. Temporal reliability correlations for a THINC‐it composite score are also reported in Table 4.

**Table 4:** Reliability and stability data for the five THINC‐it test measures (95% CI)

Task (measure)	Temporal reliability 1 (V1_A1 to V2)	Temporal reliability 2 (V1_A1 to A2)	Temporal reliability 3 (V1_A3 to V2)	Convergent validity	Stability as WSD^a^
Spotter (mean latency for correct responses)	0.79 (0.7–0.86)	0.90 (0.85–0.93)	0.86 (0.8–0.9)	0.44 (0.27–0.59)	73.5 (66.3–80.7)
Symbol Check (number correct)	0.74 (0.64–0.82)	0.68 (0.56–0.78)	0.88 (0.83–0.92)	0.19 (−0.01–0.37)	5.9 (5.3–6.5)
Codebreaker (number of correct responses)	0.81 (0.73–0.87)	0.84 (0.77–0.89)	0.80 (0.72–0.86)	0.63 (0.49–0.74)	11.23 (10.1–12.3)
Trails (time taken)	0.75 (0.48–1.1)	0.54 (0.38–0.66)	0.82 (0.74–0.88)	0.74 (0.64–0.82)	17.3 (15.6–19.0)
THINC‐it composite	0.91 (0.87–0.94)	0.93 (0.91–0.95)	0.94 (0.91–0.96)	0.42 (0.24–0.58)^c^	0.81
PDQ‐5‐D (score)^b^	0.72 (0.6–0.8)	0.72 (0.6–0.8)	0.78 (0.66–0.83)	0.92 (0.88–0.95)	0.76^d^ (0.68–0.83)

a Within‐subjects standard deviation.

b Perceived Deficits Questionnaire—Depression, 5‐item version for depression.

c (P&P)/0.752 (PDQ‐20).

d For PDQ “internal consistency.”

*Test stability* was determined by calculating the WSD value for each of the THINC‐it measures across Assessments 1 to 3 conducted at Visit 1. To quantify stability, we calculated the WSD for all four tests. Test–retest correlations, WSD, and convergent validity for all five THINC‐it components are reported in Table 4.

Correlation of THINC‐it test performance with comparison tasks to determine *convergent validity* yielded correlations between 0.19 for Symbol Check and the OBK test to 0.74 for correlations between Trails and TMT‐B. Full details of convergent validity correlations with standard error estimates are shown in Table 4.

The third element of reliability assessed was *intrarater reliability*. Empirical research has indicated that benefits of test exposure tend to accrue between the first two assessments (Falleti, Maruff, Collie, & Darby, 2006). Based on this assumption, we supposed that much of the variability attributable to test familiarity, practice, etc. would be extinguished by the second assessment. We chose therefore to correlate V1_A2 performance with that collected at V1_A3 to obtain an estimate of intrarater reliability. Levels of intrarater reliability were observed for all tests to yield values of between 0.7 and 0.93. Specific values for each test are reported in Table 3. The only THINC‐it task that lends itself to analysis of internal consistency, the PDQ‐5‐D, yielded a Cronbach's alpha score of 0.76.

## 4 DISCUSSION

In this paper, we examined in 100 typical volunteers without a history of mental disorders, levels of temporal reliability, and test stability of THINC‐it, a novel screening tool for cognitive impairment in depression. The assessment of temporal reliability over a 7‐day period (Visits 1 and 2) yielded acceptable to good test–retest reliability correlations varying between *r* = 0.74 (Symbol Check) and *r* = 0.81 (Codebreaker). Correlations increased to levels of 0.8 and above for all tests when reliability was calculated between the third assessment of Visits 1 and 2, respectively. This reliability might be indicative of a modest effect of familiarity with the tests. The reported levels exceed the threshold for 0.7 specified for acceptable temporal reliability specified by those working in psychometry (e.g., Kline, 2000, p. 26). The same threshold of 0.7 is also typically regarded as the minimum acceptable level of internal consistency (Kline, 2000, p. 28), which was observed for the self‐report PDQ‐5‐D questionnaire. The observed Cronbach's alpha score of 0.76 is also below the level at which tests might be a “bloated specific,” which can occur when the test items tend to measure essentially the same construct.

Although the primary ambition of developing THINC‐it is to provide a cognition screening instrument, we have also sought to imbue the system with test characteristics that would facilitate the evaluation of cognitive change in group studies, and potentially also in individuals. Such an approach has been advocated for some time (Harrison & Maruff, 2008), and emphasis has been placed on the need for the use of reliable measures (Harrison, 2016). The use of this methodology relies on the calculation of a Reliable Change Index (RCI). A variety of methods have been proposed for determining RCI values, and most methodologies include reliance on measures of temporal reliability (Jacobson & Truax, 1991). The examination of test stability for the THINC‐it measures, in terms of stability of repeated measures across very short intervals, in our study in a consecutive fashion, revealed acceptable levels of temporal reliability that suggest THINC‐it measures will prove useful measures of cognitive change using these approaches.

A further method for determining RCI relies on test stability, whereby Test 1 performance is plotted with a CI, determined using the WSD. This statistic is calculated using the performance of study participants on consecutive assessments, such as was conducted in our study. Later performance can then be compared against the limits of the Test 1 CI to determine statistically whether the change is a real change (i.e., outside the CI) or due to chance (i.e., inside the CI). THINC‐it tests in our validation study yielded relatively low WSD values. This is a key issue, as if WSD values are too high the resultant CI can include out of range values. Thus, a lack of reliability places significant restrictions on the utility of this approach. The debate about the utility of different RCI approaches is ongoing (see Hinton‐Bayre, 2011). We will therefore incorporate a variety of methods into THINC‐it so as to allow users to select their own preferred method of judging individual patient score change.

Our study also investigated the convergent validity of the THINC‐it tests. This was an important aspect of our investigations, as we wished to determine the extent to which THINC‐it tests performance converged with other computerized and “paper‐and‐pencil” measures of the targeted cognitive constructs of attention, working memory, and executive function. It must be noted that Choice Reaction Time, the One‐Back Task, and TMT‐B are in no sense what Kline (2000) titles “benchmark” measures of these cognitive constructs (p. 32). He proposes that in these circumstances “all that can be expected is a modest positive correlation of about .3 to .5” (p. 32). Convergent validity varied between 0.19 and 0.74 for our putative specific measures of individual cognitive constructs. The level of convergent validity observed between our general measure, “Codebreaker,” and DSST, a traditional general test of cognitive function, was 0.63, suggesting that Codebreaker scores are a reasonable proxy measure of DSST performance. Similarly, the correlation of 0.74 between THINC‐it Trails and TMT‐B indicates that the former is a robust proxy measure of the latter. However, the convergent validity between the Symbol Check task and the CogState One‐Back test was considerably lower (*r* = 0.19). One possible reason for this is the difference in task demands. The typical One‐Back Task requires a binary “yes” or “no” decision and response depending on whether the current stimulus is the same as that presented on the previous trial. Symbol Check requires the study participant to respond by touching the previous symbol, a choice of five possibilities. This typically requires the study participants to rapidly switch their attention between the stimulus sequence and the response options. In contrast, the traditional binary decision version does not typically require visual attention to the possible responses. It seems likely that Symbol Check taxes attentional and executive resources to a greater degree than traditional versions of the One‐Back paradigm. The relative lack of validity suggests that this task is not a robust proxy measure of the standard One‐Back Task, in our study exemplified by the CogState version.

A third element of test reliability in our study was our investigation of intrarater reliability. This analysis yielded reliability scores for all four tests exceeding the usual minimum acceptable level of 0.7. Intrarater reliability for the THINC‐it tests varied between a score of 0.7 for the Trails test and a high of 0.93 for the “Spotter” task.

A possible limitation of the study is that the volunteer cohort was well educated (mean YoE = 16.3, *SD* = 2.7), with a mean estimated full‐scale IQ of almost 12‐points above the population mean. Performance on cognition tests is influenced by both these factors. For example, Mitrushina et al. (2005, p. 653) suggest a minus 6.45 second subtraction from standard norm values per extra year of education for every year over 14 years for the TMT‐B. In our study, the mean TMT‐B score on first assessment was 78 seconds. The meta‐analysis of TMT‐B performance by age provided by Mitrushina et al. (2005) varies from a mean score of 54 seconds for 16‐ to 29‐year‐old study participants, and a score of 105 at the top of our age range. The 40‐ to 44‐year‐old cohort is the closest to the mean age of our sample (mean age = 40, *SD* = 14.38), and for this age group, the reported mean score is 65 seconds. This is substantially faster than our group, who at first assessment scored a mean of 78 seconds. However, it must be recalled that in our study Part A of the TMT was not administered. It seems likely that completion of Part A has a facilitative effect on Part B completion, and this may account for the observed difference. A further issue to be taken into consideration is that study participants were recruited using social media. While social media platforms are commonly utilized by all sections of the general public, it might be that respondents are among those individuals who are most comfortable with using digital technology.

In summary, this validation study of THINC‐it has shown the selected measures to be temporally reliable, to exhibit expected levels of convergent validity, and high levels of intrarater reliability and test stability. These observations support the use of THINC‐it as a brief cognitive testing system with the potential to be employed as a robust measure of cognitive change.

## DECLARATION OF INTEREST STATEMENT

In addition to receipt of funding from Lundbeck for the present study, Dr. McIntyre has received grants and personal fees from Lundbeck, Pfizer, AstraZeneca, JanssenOrtho, Purdue, Otsuka, Shire, Allergan; and personal fees from Eli‐Lilly, Johnson & Johnson, Moksha8, Sunovion, Mitsubishi, Takeda, Forest, and Bristol‐Myers Squibb.

Dr. Baune is a member of the advisory boards of Lundbeck and Janssen‐Cilag and has received honoraria from Lundbeck, Otsuka, Janssen‐Cilag, Servier, AstraZeneca, Pfizer, Wyeth, Bristol‐Myers Squibb.

Dr. Bowie is a consultant for Lundbeck, Takeda, Otsuka, and Boerhinger Ingelheim and has received grant support from Pfizer, Takeda, and Lundbeck.

Miss Cha has received royalties from Oxford University Press and Cambridge University Press.

Miss Subramaniapillai reports personal fees from Institut La Conference Hippocrate.

Dr. Culpepper has served as an advisor or consultant for Alkermes, Lundbeck, Merck, and Sunovion. Dr. Culpepper owns stock in M‐3 Information, LLc, and has received royalties from UpToDate and Oxford University Press in addition to receiving payment from Physicians Postgraduate Press as Editor in Chief of the Primary Care Companion for CNS Diseases.

Dr. Fossati has received grants from Servier and honoraria from Servier, Janssen, and Lundbeck.

Dr. Greer has received research funding from NARSAD and honoraria and/or consulting fees from H. Lundbeck A/S and Takeda.

Dr. Harmer has received consultancy fees from Lundbeck, P1vital, Ono Pharmeceuticals, and Johnson & Johnson, as well as grant funding from UCB, Johnson & Johnson, Lundbeck, and Sunovion.

Dr. Lam has received speakers fees from AstraZeneca, Canadian Network for Mood and Anxiety Treatments, Canadian Psychiatric Association, Lundbeck, Lundbeck Institute, and Otsuka; consultancy fees from Allergan, Asia‐Pacific Economic Cooperation, Bristol Myers Squibb, Canadian Depression Research and Intervention Network, Canadian Network for Mood and Anxiety Treatments, Janssen, Lundbeck, Medscape, Pfizer, Takeda; research funds from BC Leading Edge Foundation, Brain Canada, Bristol Myers Squibb, Canadian Institutes of Health Research, Canadian Depression Research and Intervention Network, Canadian Network for Mood and Anxiety Treatments, Janssen, Lundbeck, Movember Foundation, Pfizer, St. Jude Medical, University Health Network Foundation, Vancouver Coastal Health Research Institute, VGH Foundation. In addition, Dr. Lam owns the copyright to the Lam Employment Absence and Productivity Scale (LEAPS).

Dr. Mansur reports fellowship funding from Lundbeck outside the submitted work.

Dr. Wittchen reports honoraria from Lundbeck.

Dr. Harrison reports personal fees from Lundbeck during the conduct of the study; personal fees from AbbVie, Amgen, Anavex, AstraZeneca, Avonex, Avraham, Axon Neuroscience, Axovant, Biogen Idec, Boehringer Ingelheim, Bracket, Catenion, CRF Health, DeNDRoN, Eisai, Eli Lilly, EnVivo Pharma, Enzymotec, ePharmaSolutions, Forum Pharma, GfHEU, Heptares, Janssen AI, Johnson & Johnson, Kaasa Health, Kyowa Hakko Kirin, MedAvante, Merck, Mind Agilis, MyCognition, Neurim, Neurocog, Neurotrack, Novartis, Nutricia, Orion Pharma, Pfizer, Pharmanet/i3, Prana Biotech, PriceSpective, Probiodrug, Prophase, Prostrakan, Regeneron, Reviva, Roche, Sanofi, Servier, Takeda, TransTech Pharma, and Velacor. In addition, Dr. Harrison has a patent Cognition training system pending to MyCognition.

Dr. Barry, Dr. Best, Miss Carmona, Miss Lee, and Dr. Klag have no conflicts to disclose.
